# How Do Patient Demographics and Socioeconomic Disadvantage Impact Clinical Presentation, Surgical Outcomes, and Survival for Upper Extremity Soft Tissue Sarcoma?

**DOI:** 10.1002/cnr2.70445

**Published:** 2026-01-14

**Authors:** Nicole J. Newman‐Hung, Kameel Khabaz, Michaela Juels, Giovanni Gamalong, Daniel Chiou, Bailey Mooney, Nicholas M. Bernthal, Lauren E. Wessel

**Affiliations:** ^1^ Department of Orthopaedic Surgery University of California Los Angeles California USA; ^2^ David Geffen School of Medicine, University of California Los Angeles California USA; ^3^ Department of Orthopaedic Surgery Harbor‐UCLA Medical Center Torrence California USA

**Keywords:** socioeconomic disadvantage, soft tissue sarcoma, upper extremity cancers

## Abstract

**Background:**

Soft tissue sarcomas (STS) of the upper extremity (UE) are uncommon and may require complex surgical management. Socioeconomic disadvantage, race/ethnicity, sex, and marital status may influence presentation, surgical complexity, local recurrence (LR), and overall survival (OS).

**Aims:**

The aim of this work is to examine the influence of socioeconomic and demographic factors on the presentation and outcomes of upper extremity soft tissue sarcomas.

**Methods and Results:**

We identified patients treated surgically for primary UE STS (2012‐2022). Demographics, tumor characteristics, and outcomes were recorded. Associations between demographics and time to presentation, skin grafting, amputation, and LR were assessed using chi‐square and *t*‐tests. Competing risks regression analyzed 3‐ and 5‐year LR, and Kaplan‐Meier analysis assessed 5‐year OS. Among 181 patients, the mean time to presentation was 17.1 months (SD 34.3), mean tumor size was 7.8 cm (SD 5.7) while 56% required re‐excision, 15% underwent amputation, 15% required skin grafting, and 24% experienced LR. Hispanic/Latino patients presented with larger tumor sizes (9.17 ± 5.71 cm vs. 7.43 ± 5.61 cm, *p* = 0.037). Non‐married patients had higher odds of amputation (OR 3.15, *p* = 0.012), and female sex predicted greater LR risk (OR 2.17, *p* = 0.037). Twenty‐one patients (11.1%) died within five years. In multivariable analysis, increasing tumor size (OR = 1.08; *p* = 0.010) and high tumor grade (OR = 8.28; *p* = 0.038) significantly impacted 5‐year OS.

**Conclusion:**

While disparities across patient demographics may exist for surgical outcomes for UE STS, racial disparities in overall survival may be mitigated with treatment at an urban, tertiary care sarcoma center.

## 1 Introduction

Soft tissue sarcomas (STS) are rare tumors with potentially devastating effects on patients [1]. Surgical resection and radiotherapy are the mainstay of management, but rates of local recurrence (LR) remain persistently high, ranging from 13% to 39% [1, 2, 3, 4, 5, 6]. Patients who develop LR often have worse functional outcomes, higher rates of distant metastases, and decreased overall survival (OS) [2, 3, 4, 7, 8]. In addition, STS may mimic benign inflammatory lesions or melanoma, so misdiagnosis may lead to unplanned excisions with positive margins and poor long‐term outcomes [9, 10]. Upper extremity (UE) tumors represent 15% of all STS [11, 12]. Tumors in the UE present distinct surgical considerations due to the narrower margin for resection afforded by their proximity to critical neurovascular structures [13, 14, 15]. As a result, discussions of soft tissue coverage requirements and limb salvage versus amputation are critical aspects of preoperative counseling and patient counseling [14, 15, 16].

While tumor and treatment‐related predictors of surgical outcomes, LR, and OS have been described, the impact of patient demographics and socioeconomic disadvantage on these outcomes remains elusive [2, 6]. Furthermore, racial and insurance‐based disparities in oncologic outcomes have been described across various cancer types, but it is unknown if racial disparities persist throughout UE STS outcomes [17, 18]. Deepening our understanding of how patient factors and social environment may impact surgical and oncologic outcomes for rare diseases such as UE STS is critical to improving access to care for all patients.

This study uses the largest contemporary surveillance database of patients with primary UE STS to analyze the impact of demographic and socioeconomic factors on time to orthopaedic oncologic consultation, presenting tumor characteristics, and post‐operative rates of local recurrence and overall survival.

## 2 Methods

### 2.1 Level of Evidence: III

#### 2.1.1 Study Design and Setting

This study was performed at the University of California, Los Angeles, an urban, high‐volume tertiary care sarcoma center. We retrospectively reviewed all primary UE STS cases treated surgically at our institution from January 1, 2012, to December 31, 2022.

#### 2.1.2 Patients

We retrospectively queried our institution's UE STS database for patient demographics, presenting tumor characteristics, treatment details, and outcomes. UE tumors were defined as those arising from shoulder girdle proximally to fingertip distally. We included all patients with primary disease who were treated surgically from January 1, 2012, to December 31, 2022. Additionally, patients who were seen primarily as a second opinion for tumor board presentation and those with limited clinical follow‐up were excluded.

#### 2.1.3 Baseline Data

A total of 190 patients met inclusion criteria with 112 (59%) males and 78 (41%) females. In terms of race, 118 patients (62%) were White, 33 (17%) were Other (self‐identified), 22 (12%) were Asian/Pacific Islander, and 12 (6%) were Black. With respect to ethnicity, 147 patients (77%) were non‐Hispanic and 41 (22%) were Hispanic. Race and ethnicity were not available in a small subset of patients (3% for race and 1% for ethnicity). One hundred six patients (56%) were married while 84 (44%) were non‐married. Average ADI of our cohort was 17.1. Complete demographic data are shown in Table 1.

**TABLE 1:** Patient demographics.

Age at presentation, median (years)	52.0 (IQR: 38.0–68.0)
Sex, *n* (%)	
Male	112 (59)
Female	78 (41)
Race, *n* (%)	
White	118 (62)
Other	33 (17)
Asian/Pacific Islander	22 (12)
Black	12 (6)
N/A	5 (3)
Ethnicity, *n* (%)	
Non‐Hispanic	147 (77)
Hispanic	41 (22)
N/A	2 (1)
Marital status, *n* (%)	
Married	106 (56)
Non‐married	84 (44)
Area deprivation index (ADI)	17.1 (IQR: 6.0–24.0)

#### 2.1.4 Variables and Data Sources

Patient data were extracted from the electronic medical record and recorded in our institution's UE STS surveillance database. Variables recorded from the database included patient sex, race, ethnicity, insurance, zip code, marital status, employment status, age at time of presentation, duration of symptoms prior to presentation, initial biopsy date, initial biopsy type, tumor location, histopathologic diagnosis, tumor grade, preoperative and postoperative tumor size, tumor depth (superficial vs. deep to fascia), date of surgery, surgeon specialty (orthopaedic oncology versus surgical oncology versus general surgery vs. hand surgery), surgery location (sarcoma center vs. outside hospital), nature of excision (unplanned versus planned), and margin status (negative, close, or positive). Date of death and/or last date of clinical follow‐up were recorded.

Socioeconomic deprivation was quantified with Area Deprivation Index (ADI), derived from University of Wisconsin's validated Neighborhood Atlas using patients' zip codes [19]. Patients were assigned national ADIs from 1 to 100, with 100 indicating the highest level of deprivation. ADI is generated from 17 metrics reflecting housing, education, income, education, and employment [19]. Patients were then divided into upper three quartiles versus lowest quartile (least deprived) for analysis.

#### 2.1.5 Primary and Secondary Study Outcomes

Primary outcome measures were 3‐ and 5‐year LR and 5‐year overall survival. Secondary outcome measures included time to presentation, tumor size at presentation, skin graft requirement, and amputation.

#### 2.1.6 Statistical Analysis

Continuous and categorical variables were reported as median and interquartile range (IQR), or as frequency (%), respectively. In univariate analysis, the significance of inter‐cohort differences was evaluated using a chi‐square test for categorical variables and Mann–Whitney U test for continuous variables. Given low event rates for secondary outcome measures of skin grafting and amputation, multivariable analysis was not performed.

Three‐ and 5‐year LR risks were evaluated with the Fine and Gray competing risks approach [20]. Univariate and multivariate analyses were performed to identify predictors for OS. Five‐year OS of the entire cohort was plotted as a survival curve based on the Kaplan–Meier estimator, with 95% confidence intervals [21].

## 3 Results

### 3.1 Time to Presentation

Overall mean time to presentation was 17.1 months (SD 34.3). Overall mean presenting tumor size was 7.8 cm (SD 5.7). Mean time to presentation was similar across sex, race, marital status cohorts, and ADI quartiles; however, Hispanic/Latino ethnicity was associated with larger tumor size upon presentation (9.17 ± 5.71 cm vs. 7.43 ± 5.61 cm, *p* = 0.037). Data are displayed in Table 2.

**TABLE 2:** Presenting characteristics and surgical outcomes stratified by patient demographics and Area Deprivation Index.

	Sex			Race			Ethnicity			Marital status			ADI		
	M^+^	F	*p* OR	W^+^	NW	*p* OR	N‐H/L^+^	H/L	*p* OR	M^+^	NM	*p* OR	Upper Three Quartiles^+^	Lowest Quartile	*p* OR
Mean time to presentation in months	16.11 ± 29.30	18.50 ± 40.29	0.518	17.05 ± 38.73	17.00 ± 24.83	0.294	16.70 ± 34.68	19.04 ± 33.05	0.316	13.49 ± 26.43	21.53 ± 41.41	0.124	17.93 ± 35.27	16.66 ± 34.10	0.496
Mean tumor size at presentation in centimeters	7.97 ± 6.33	7.44 ± 4.52	0.963	7.76 ± 5.83	7.68 ± 5.53	0.706	7.43 ± 5.61	9.17 ± 5.71	**0.037**	8.18 ± 6.25	7.21 ± 4.75	0.363	8.05 ± 4.57	7.80 ± 5.97	0.301
Graft required, *n* (%)	19 (17%)	10 (13%)	0.564 0.72	20 (18%)	7 (10%)	0.230 0.52	18 (12%)	10 (24%)	0.092 2.31	17 (16%)	12 (14%)	0.896 0.87	9 (20%)	18 (13%)	0.309 0.57
Amputation, *n* (%)	14 (12%)	14 (18%)	0.404 1.53	21 (19%)	7 (10%)	0.179 0.49	22 (15%)	6 (15%)	1.000 0.97	9 (8%)	19 (23%)	**0.012** 3.15	4 (9%)	22 (16%)	0.403 1.85
Local recurrence, *n* (%)	20 (18%)	25 (32%)	**0.037** 2.17	33 (29%)	11 (16%)	0.060 0.45	40 (27%)	5 (12%)	0.074 0.37	27 (25%)	18 (21%)	0.632 0.80	9 (20%)	34 (24%)	0.766 1.24

*Note:* *ADI = Area Deprivation Index; F = female; H/L = Hispanic/Latino; M = male; M = married; N‐H/L = Non‐Hispanic/Latino; NM = non‐married; NW = nonwhite; OR = odds ratio; W = white; ^+^ = reference group for OR.

### 3.2 Surgical Outcomes

Twenty‐eight patients (15%) underwent amputation. Non‐married patients (23%) were more likely to require amputation compared to married patients (8%) (OR 3.15, *p* = 0.012). Twenty‐nine patients (15%) required skin grafting. There were no differences in graft requirements across all demographic groups.

**FIGURE 1:**
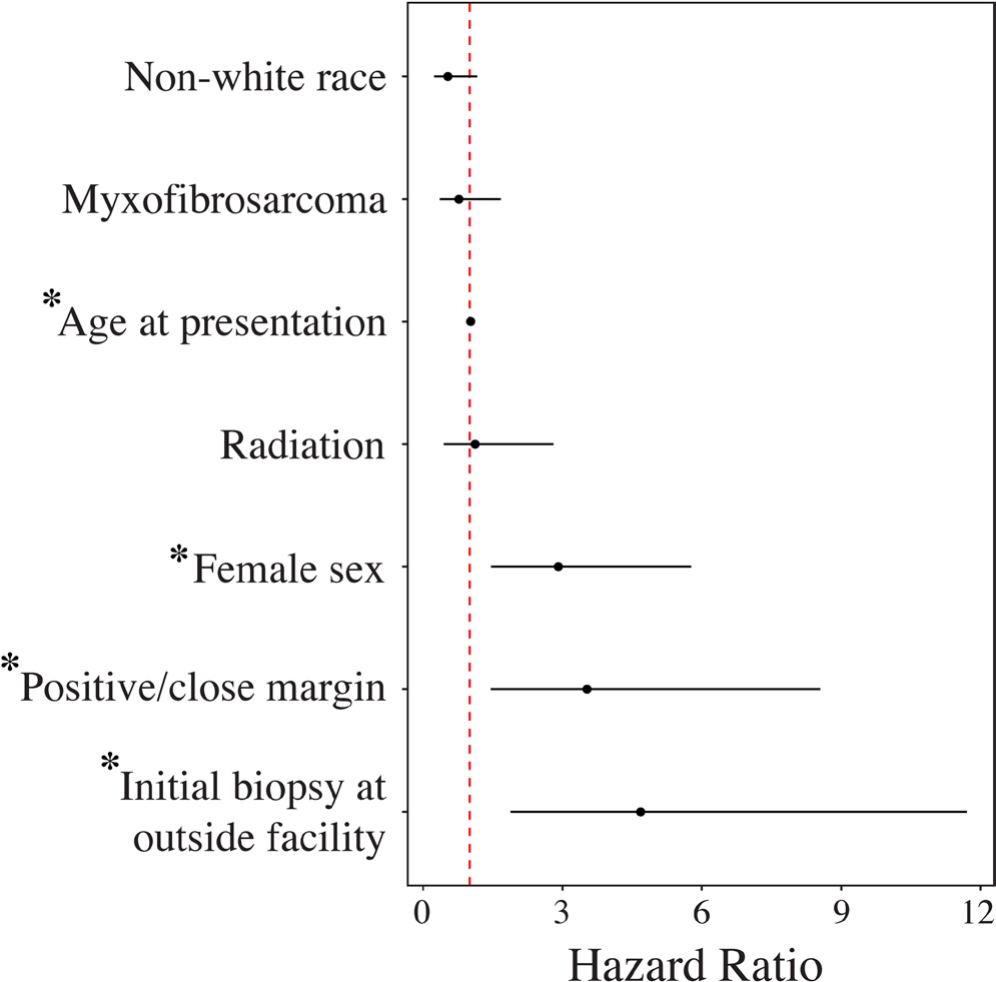
Subdistribution Hazard Ratio Estimates for Risk Factors Associated with 3‐year LR (95% Confidence Interval). *p < 0.05.

Forty‐five patients (24%) experienced LR. In univariate analysis, female sex (32%) was associated with a higher rate of LR compared to male sex (18%) (OR 2.17, *p* = 0.037). There were otherwise no demographic or ADI‐based differences in LR. In competing risks models for 3‐ and 5‐year LR evaluating sex and race while incorporating tumor and treatment factors, female sex remained a risk factor for LR after adjusting for covariates (OR 2.91, *p* = 0.002). A breakdown of histologic subtype by sex, shown in Table 3, demonstrates no significant subtype clustering (*p* = 0.960). Nonwhite race was not a significant risk factor. Increasing patient age (OR 1.02, *p* = 0.03), undergoing initial excision at a non‐sarcoma center (OR 4.69, *p* < 0.001), and undergoing R1 or R2 resection (OR 3.53, *p* = 0.005) were significantly associated with LR. Subdistribution hazard ratio (HR) estimates for 3‐year LR are shown in Figure 1. The 5‐year LR HR did not significantly differ from that of 3‐year LR.

**TABLE 3:** Histologic subtype by sex.

	Male (*N*, %)	Female (*N*, %)
Myxofibrosarcoma	13 (16.7%)	18 (16.1%)
Undifferentiated pleomorphic sarcoma	13 (16.7%)	18 (16.1%)
Undifferentiated sarcoma	12 (15.4%)	16 (14.3%)
Liposarcoma	8 (10.3%)	12 (10.7%)
Synovial sarcoma	10 (12.8%)	9 (8.0%)
Leiomyosarcoma	3 (3.8%)	10 (8.9%)
Undifferentiated spindle cell sarcoma	4 (5.1%)	5 (4.5%)
Epithelioid sarcoma	3 (3.8%)	5 (4.5%)
Dermatofibrosarcoma protuberans	2 (2.6%)	4 (3.6%)
Other	10 (12.8%)	15 (13.4%)

### 3.3 Overall Survival

**FIGURE 2:**
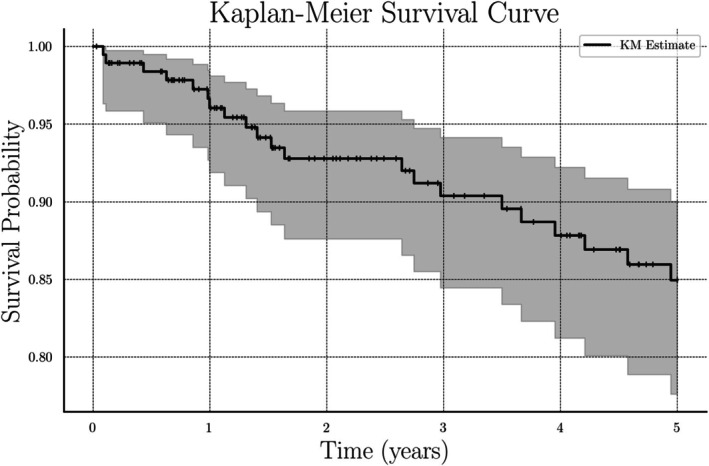
Kaplan Meier curve for 5‐year OS (95% Confidence Interval).

Five‐year OS was 88.9% as 21 patients (11.1%) died within 5 years of their index surgery. In univariate analysis, there were no differences in 5‐year OS based on sex, race, marital status, primary language, insurance status, employment status, or ADI quartile. Similarly, in multivariable analysis, patient race and ADI did not significantly impact OS. Increasing tumor size (OR = 1.08; *p* = 0.010) and high tumor grade (OR = 8.28; *p* = 0.038) were significant independent risk factors impacting 5‐year OS. Complete univariate and multivariable analyses for OS are demonstrated in Tables 4 and 5. Kaplan Meier curve for 5‐year OS is shown in Figure 2.

**TABLE 4:** Univariate analysis of factors impacting 5‐year overall survival.

Univariate analysis	HR	95% CI	*p*
Age at time of presentation, per ten‐year increase	1.014	(0.992, 1.037)	0.221
Female sex	0.597	(0.231, 1.538)	0.285
Non‐white race	1.632	(0.693, 3.844)	0.263
Non‐married	0.871	(0.361, 2.101)	0.758
Non‐English primary language	0.327	(0.044, 2.437)	0.275
Medicaid Insurance	0.931	(0.217, 3.998)	0.923
Not working	1.372	(0.568, 3.311)	0.482
ADI Upper Three Quartiles	0.395	(0.092, 1.699)	0.212
Time from symptoms to presentation, per 1 month increase	0.955	(0.905, 1.009)	0.1
Tumor size, per 1 cm increase	**1.056**	**(1.017, 1.097)**	**0.005**
High grade tumor	3.226	(0.751, 13.851)	0.115
Initial biopsy at outside facility	**10.493**	**(2.44, 45.129)**	**0.002**
Unplanned excision	**0.225**	**(0.076, 0.671)**	**0.007**
Radiation	0.551	(0.228, 1.33)	0.185
Systemic therapy	2.097	(0.89, 4.94)	0.09
Positive/close final margin status	1.014	(0.992, 1.037)	0.221

*Note:* Bold values indicate statistical significance (*p* < 0.05).

**TABLE 5:** Multivariable analysis of factors impacting 5‐year overall survival.

Multivariate Cox Regression	HR	95% CI	*p*
Age at time of presentation, per 10‐year increase	1.08	(0.86, 1.35)	0.528
Nonwhite race	2.20	(0.87, 5.57)	0.096
ADI bottom quartile (least deprived)	3.06	(0.70, 13.41)	0.138
Tumor size, per 1 cm increase	1.08	(1.02, 1.14)	**0.010**
High grade tumor	8.28	(1.12, 61.12)	**0.038**
Positive/close final margin status	2.50	(0.65, 9.61)	0.181

*Note:* Bold values indicate statistical significance (*p* < 0.05).

## 4 Discussion

Before proposing concrete measures to improve equity in STS care, we must deepen our understanding of how different aspects of patient demographics are associated with worse access to care and inferior outcomes. Prior studies utilizing national cancer databases have demonstrated disparities in both access to sarcoma care and in oncologic outcomes [22, 23, 24, 25]. While beneficial in adequately powering analysis, registry studies may be limited in the granularity of patient demographics and surgical/oncologic outcomes available for analysis. In this study, we leveraged our institutional sarcoma surveillance database to present the largest single‐center contemporary cohort of UE STS cases. We report disparities across patient sex, race/ethnicity, and marital status; surgical outcomes, LR, and OS. Contrary to national database studies, there were no differences across patient demographics in time to presentation. Furthermore, socioeconomic disadvantage did not impact time to presentation or surgical or oncologic outcomes.

Race and insurance‐based disparities in access to care remain prevalent throughout sarcoma management [18, 25, 26, 27]. Insurance status has been reported to impact disease progression at the time of presentation across various cancer types. In an analysis of the top 10 most deadly cancers recorded in the SEER database, Walker et al. reported that patients with non‐Medicaid insurance were less likely to present with metastatic disease (16.9% vs. 29.1% for Medicaid) [18]. Diessner et al. reported that patients with Medicaid or uninsured insurance status with STS, not bone sarcoma, were more likely to present with metastases [26]. On the other hand, Hu et al. demonstrated that patients with Medicaid and uninsured patients with bone sarcoma also were more likely to present with metastatic disease [27].

Rather than assessing disease progression at time of presentation, we examined mean time to presentation in months, reflecting time from symptom onset to orthopaedic oncology consultation. Contrary to registry studies, we did not find any racial, insurance, or socioeconomic‐based differences in time to presentation [28]. However, we found ethnicity‐based differences in tumor size upon presentation, with Hispanic/Latino patients presenting with significantly larger tumors than non‐Hispanic/Latino patients. While Hispanic/Latino patients did not experience significantly longer time to presentation in our cohort, barriers to promptly seeking formal medical care for concerning soft tissue tumors may still exist. For example, Hispanic/Latino patients may have lower general awareness of sarcoma‐related symptoms [29]. Literature from other medical subspecialties has demonstrated that language barriers and systemic mistrust of the medical community may also impede patients from seeking formal evaluation [30, 31]. These factors may also contribute to larger tumor size at time of presentation.

Ethnicity‐based disparities in limb salvage have also been reported, as registry studies have demonstrated higher amputation rates in Hispanic patients with extremity STS compared to non‐Hispanic patients after controlling for tumor size (24% lower limb‐salvage rate) [28]. We did not find any racial disparities in limb salvage rates; however, we found a significant association between non‐married marital status and increased amputation risk, as non‐married patients were over 3 times more likely to undergo amputation. The impact of marital status specifically on amputation rates has not been investigated in registry databases. However, in a SEER analysis of extremity STS patients, Alamanda et al. found that non‐married patients received radiotherapy at lower rates compared to married patients [32]. Marital status has also been shown to impact survivorship in cancer patients who undergo amputation [33]. Psychosocial support may also encourage sarcoma patients to seek initial consultation and maintain appropriate surveillance schedules. Social support has been shown to impact functional outcomes and patient satisfaction following both limb salvage and amputation [34].

We also identified modifiable and non‐modifiable risk factors for 3‐ and 5‐year LR, including female sex, increasing age, undergoing R1/R2 resection, and undergoing initial resection at a non‐sarcoma center. Our 5‐year LR rate of 26% was consistent with rates reported from prior institutional studies, particularly those from sarcoma centers and tertiary referral institutions [35, 36, 37]. Contrary to prior studies, we identified female sex as an independent risk factor for 3‐ and 5‐year LR. Interestingly, there were no racial, ethnic, or socioeconomic disadvantage‐based disparities in LR rates as previously reported [38]. Our unique findings suggest that these differences may be attenuated when receiving care at an urban tertiary care center. Another explanation for this may be that there were no differences in rates of adjuvant radiotherapy between these patient cohorts in contrast to prior registry studies, which showed differences in management based on socioeconomic disadvantage [23].

Most notably, we did not find race or ethnicity‐based disparities in 5‐year OS. These findings stand in stark contrast to those of prior national registry studies demonstrating worse survivorship in extremity STS for Black patients [22, 24]. Similarly, we did not find any differences in 5‐year OS between insurance cohorts whereas Walker et al. also reported that Medicaid insurance was associated with worse OS for the top 10 most deadly cancers [18]. Similar to the discussion on LR, attenuation of disparities in OS may be associated with our findings of no significant differences in adjuvant radiotherapy or chemotherapy administration between racial and ethnic groups at our institution. Ultimately, these findings suggest that racial and insurance‐based disparities may be mitigated but not eliminated when seeking sarcoma care from a large, urban academic tertiary care center.

## 5 Conclusions

In this study, we report differences in surgical outcomes with attenuation of disparities in 3‐ and 5‐year LR and 5‐year OS between patient demographic and socioeconomic groups. For UE STS patients receiving care at a high‐volume urban sarcoma center, the racial, ethnic, and socioeconomic survival gaps routinely observed in national registries are markedly reduced, suggesting that timely referral to specialized multidisciplinary programs may mitigate underlying disparities. The higher amputation risk in non‐married patients and elevated local recurrence risk in women highlight the importance of proactive engagement in these patient populations.

## Author Contributions

**Nicole J. Newman‐Hung:** conceptualization (equal), data curation (equal), formal analysis (equal), methodology (equal), writing – original draft (equal), writing – review and editing (equal). **Kameel Khabaz:** data curation (equal), formal analysis (equal), methodology (equal), writing – original draft (equal), writing – review and editing (equal). **Michaela Juels:** data curation (equal), writing – review and editing (equal). **Giovanni Gamalong:** data curation (equal), writing – review and editing (equal). **Daniel Chiou:** conceptualization (equal), data curation (equal). **Bailey Mooney:** conceptualization (equal), data curation (equal). **Nicholas M. Bernthal:** conceptualization (equal), project administration (equal). **Lauren E. Wessel:** conceptualization (equal), project administration (equal), resources (equal), writing – original draft (equal), writing – review and editing (equal).

## Funding

This work was supported by the National Institute of Arthritis and Musculoskeletal and Skin Diseases (T32AR059033‐12).

## Ethics Statement

We obtained institutional review board approval for this study (IRB #23‐001022).

## Conflicts of Interest

N.B. reports funding from Deciphera Pharm LLC, unrelated to this work. All other authors certify that there are no funding or commercial associations (consultancies, stock ownership, equity interest, patent/licensing arrangements, etc.) that might pose a conflicts of interest in connection with the submitted article related to the author or any immediate family members.

## Data Availability

De‐identified data are available from the corresponding author on reasonable request.
